# Bone marrow-independent adventitial macrophage progenitor cells contribute to angiogenesis

**DOI:** 10.1038/s41419-022-04605-2

**Published:** 2022-03-09

**Authors:** Florian Kleefeldt, Berin Upcin, Heike Bömmel, Christian Schulz, Georg Eckner, Jan Allmanritter, Jochen Bauer, Barbara Braunger, Uwe Rueckschloss, Süleyman Ergün

**Affiliations:** 1 Institute of Anatomy and Cell Biology, University of Wuerzburg, Koellikerstraße 6, 97070 Wuerzburg, Germany; 2 Medizinische Klinik und Poliklinik I, Klinikum der Universität, Ludwig-Maximilians-Universität, Marchioninistrasse 15, 81377 Munich, Germany

**Keywords:** Stem-cell differentiation, Stem-cell niche, Adult stem cells, Cell signalling

## Abstract

Pathological angiogenesis promotes tumor growth, metastasis, and atherosclerotic plaque rupture. Macrophages are key players in these processes. However, whether these macrophages differentiate from bone marrow-derived monocytes or from local vascular wall-resident stem and progenitor cells (VW-SCs) is an unresolved issue of angiogenesis. To answer this question, we analyzed vascular sprouting and alterations in aortic cell populations in mouse aortic ring assays (ARA). ARA culture leads to the generation of large numbers of macrophages, especially within the aortic adventitia. Using immunohistochemical fate-mapping and genetic in vivo-labeling approaches we show that 60% of these macrophages differentiate from bone marrow-independent Ly6c^+^/Sca-1^+^ adventitial progenitor cells. Analysis of the *NCX*^−/−^ mouse model that genetically lacks embryonic circulation and yolk sac perfusion indicates that at least some of those progenitor cells arise yolk sac-independent. Macrophages represent the main source of VEGF in ARA that vice versa promotes the generation of additional macrophages thereby creating a pro-angiogenetic feedforward loop. Additionally, macrophage-derived VEGF activates CD34^+^ progenitor cells within the adventitial vasculogenic zone to differentiate into CD31^+^ endothelial cells. Consequently, depletion of macrophages and VEGFR2 antagonism drastically reduce vascular sprouting activity in ARA. In summary, we show that angiogenic activation induces differentiation of macrophages from bone marrow-derived as well as from bone marrow-independent VW-SCs. The latter ones are at least partially yolk sac-independent, too. Those VW-SC-derived macrophages critically contribute to angiogenesis, making them an attractive target to interfere with pathological angiogenesis in cancer and atherosclerosis as well as with regenerative angiogenesis in ischemic cardiovascular disorders.

## Introduction

Angiogenesis, the formation of new blood vessels based on preexisting vasculature, is involved in physiological and pathological processes like wound healing, organ growth, and maintenance as well as retinopathy, cardiovascular diseases, and tumor progression [1–4]. Angiogenesis particularly occurs under hypoxic conditions, i.e., in tumor growth and ischemic cardiovascular disorders. Hypoxia upregulates the expression of vascular endothelial growth factor (VEGF) which in turn acts as a major pro-angiogenic modulator inducing endothelial cell proliferation, migration, and vascular formation [5–7]. In cancer therapy, inhibition of VEGF-mediated angiogenesis is used to decelerate disease progression [8].

The formation of new blood vessels is a complex process involving different cell types and endogenous factors that either promote or inhibit it. For a long time, the contribution of mature endothelial cells and their circulating progenitors has been the focus of angiogenesis research [9, 10]. However, during the last decades increasing attention has been paid to the impact of non-vascular cell types like macrophages [11, 12], especially in the context of tumor angiogenesis [13]. These macrophages that are abundant in tumors are called tumor-associated macrophages (TAMs) [14, 15]. TAMs were shown to produce high levels of VEGF that is the most potent pro-angiogenic molecule known so far [16, 17]. In addition, the presence of TAMs was associated with a worse prognosis, i.e., for gastric and breast cancers [18–20]. In order to elucidate the origin of TAMs, Fujimoto et al. showed augmented infiltration of circulating macrophages into the tumor due to stromal expression of macrophage chemoattractant protein-1 (MCP-1) that resulted in increased angiogenesis as well as tumor growth in a murine tumor model [21]. However, in clinical studies, tumor treatment via inhibition of monocyte recruitment was very inefficient [22–28].

Besides infiltration, pro-angiogenic macrophages could also arise by differentiation from resident progenitor cells. Vascular wall-resident stem and progenitor cells (VW-SCs) were shown to deliver vascular progenitors, but also non-vascular cells like cardiomyocytes and macrophages [29–32]. VW-SCs can be activated and mobilized from their adventitial niche due to co-culture with tumor cells further underscoring their potential to facilitate tumor angiogenesis [33–36]. However, the impact of bone marrow-independent VW-SC-derived macrophages on vascular progenitor cell activation and angiogenesis remains unclear [37–39].

To this end, we used the ex vivo murine aortic ring assay (ARA) and examined the origin of macrophages and their impact on the activation of the adventitial vasculogenic zone, on VW-SC mobilization, differentiation, and angiogenic sprouting activity. We found that the majority of F4/80^+^ macrophages differentiates from bone marrow-independent adventitial Ly6c^+^/Sca-1^+^ VW-SCs. In addition, we also identified these Ly6c^+^/Sca-1^+^ progenitor cells in the aorta of embryonic *NCX1*^−/−^ mice that lack embryonic yolk sac circulation [40, 41]. This indicates that these cells at least partially are yolk sac-independent, too. The generated macrophages become the main source of VEGF in ARA. On one hand, this VEGF promotes the generation of additional macrophages thereby creating a pro-angiogenic feedforward loop. On the other hand, macrophage-derived VEGF activates CD34^+^ progenitor cells within the adventitial vasculogenic zone to differentiate into CD31^+^ endothelial cells. Consequently, clodronate-mediated macrophage depletion as well as VEGFR2 antagonism resulted in the preservation of the adventitial VW-SC niche and reduced sprouting and capillary-like tube formation.

Our data show that it is important to consider the contribution of bone marrow-independent VW-SCs to the macrophage pool and their impact on angiogenetic processes in order to understand disease progression e.g., in cancer and atherosclerosis. Identification of triggers and mechanisms of macrophage differentiation from VW-SCs and their therapeutic manipulation may open up new strategies to impede pathologically but promote regenerative angiogenesis.

## Materials and methods

### Animals

Eight-week-old male C57BL/6 J (Jackson Laboratory, Bar Harbor, ME, USA), FlkSwitch mice [33, 34], and *NCX1*^*−/*−^ mice [35, 36] were housed in specific pathogen-free conditions on a 12:12-h dark-light cycle and fed a standard chow ad libitum. All experiments were performed according to German and American animal protection law.

FlkSwitch were obtained by breeding Flt3-Cre mice with ROSA mT/mG mice (Gt(ROSA)26Sortm4(ACTB-tdTomato,-EGFP), Jackson Laboratory). In the absence of Cre recombinase ROSA mT/mG mice constitutively express tdTomato under the control of the ubiquitous *ACTB* promoter. Cre expression in Flt3^+^ cells (e.g., bone marrow-derived monocyte progenitors) results in the excision of the tdTomato expression cassette, which restores the eGFP expression cassette in these cells. FlkSwitch mice were used to distinguish macrophages differentiated during ARA from bone marrow-derived progenitor cells (eGFP^+^) from those macrophages that differentiated from bone marrow-independent vascular resident progenitor cells (tdTomato^+^).

### Aortic ring assay

ARA was used to study angiogenic processes in vitro as described previously [42–44]. After cervical dislocation murine thoracic aortae were dissected by laparo- and thoracotomy. Perivascular fat was removed and intravascular blood was flushed out by perfusion with PBS. The thoracic aorta was cut into ring segments of 1 mm length that were embedded in a collagen gel. Collagen gel solution were made up of 4 ml PureCol^®^ collage type I solution (Advanced BioMatrix, San Diego, CA, USA), 1 ml 10x MEM, 500 µl 7.5% Na-bicarbonate, 100 µl 200 mM l-glutamine, 100 µl 100 mM Na-pyruvate, and penicillin/streptomycin solution (1:100) adjusted to pH 7.4 with 1 M NaOH and filled up to 10 ml using distilled water. MEM, l-glutamine, Na-bicarbonate were provided by Sigma-Aldrich (St. Louis, MO, USA). Na-pyruvate and penicillin/streptomycin were purchased from Thermo Fisher Scientific (Waltham, MA, USA). Aortic ring segments were cultured for 11 days in DMEM/10%FCS (Thermo Fisher/Biochrom, Berlin, Germany; 37 °C; 5% CO2). The medium was changed every third day.

Some aortic rings were treated with the specific VEGFR2 antagonist ZM323881 (1 µM; Selleck Chemicals, Houston, TX, USA). Sprouting was documented using a phase-contrast microscope (Leica DFC 3000 G, Wetzlar; Germany).

### Macrophage depletion

Macrophages were depleted by application of clodronate-containing liposomes to the collagen-embedded aortic rings (100 µM; Liposoma B.V., Amsterdam, The Netherlands) according to previously published protocols [45, 46]. Aortic rings treated with clodronate-free, PBS-containing liposomes served as controls.

### Immunohistochemical analyses

Freshly isolated aorta (FIA) and cultured aortic rings were fixed (4% PFA; 24 h, RT) and embedded in paraffin or Tissue-Tek^®^ (Sakura Finetek Europe B.V., Alphen aan den Rijn, NL). Immunohistochemistry was performed as described previously [47, 48]. Antibodies used in this study are listed in Table 1. Images were taken by a BZ9000 microscope (Keyence^®^, Biorevo, Neu-Isenburg, Germany) or NIKON Eclipse TI-E inverse confocal microscope (co-immunofluorescence) and NIS-Elements Advanced Research software (Nikon) [49]. The immunohistochemical micrographs are representative of at least three independent experiments.

**Table 1:** Antibodies used in this study.

Antigen	Manufacturer	Catalog number	Dilution
CD31	Abcam	ab28364	1:200
CD34	Abcam	ab8158	1:50
F4/80	Abcam	ab111101	1:100
Ki67	Abcam	ab16667	1:50
Ly6c	Abcam	ab15627	1:100
Sca-1	R&D Systems	AF1226	1:100
VEGF	Abcam	ab46154	1:200
VEGFR2 (Flk-1)	Santa Cruz	sc-393163	1:50

### Dispersion of aortic tissue and magnetic cell sorting (MACS) of FIA

Thoracic aortae were incubated with collagenase type 2 solution (2.5 mg/ml; Worthington Biochemical Corp., Lakewood, NJ, USA) for 3 h followed by inactivation of collagenase activity using 5% FCS/PBS. Aortic cells were suspended in MACS buffer (0.5 M EDTA, 0.6% BSA) and were incubated with an anti-Ly6c antibody (1:20; Abcam, Cambridge, UK; ab15627) at 4 °C for 20 min. Ly6c^+^ cells were separated using magnetic beads-conjugated secondary antibodies (1:4; 4 °C for 15 min; Miltenyi, Bergisch Gladbach, Germany; 130-048-502) and a MACS column (MS column, Miltenyi). Isolated Ly6c^+^ cells were cultured in DMEM/10%FCS/1% penicillin/streptomycin for 2 days at 37 °C/5% CO_2_.

### Cell viability assay

EA.hy926 endothelial cells (American Type Culture Collection, Manassas, VA, USA) were cultured in DMEM/10%FCS/1% penicillin/streptomycin at 37 °C/5% CO_2_ to confluence. Cell viability was tested after incubation with/or without clodronate-containing liposomes or PBS-containing liposomes (each 100 µM) for 48 h using the CellTiter-Glo^®^ 2.0 Cell Viability Assay (Promega Corporation, Madison, WI, USA) according to manufacturer’s instructions.

### Quantification and statistical analyses

Cell counts, aortic segment circumference, and total media area were evaluated using ImageJ 1.47 (Wayne Rasband; National Institute of Health; USA). Cell counts were normalized to total media area (given by ImageJ as arbitrary units).

Data are given as mean ± SEM. In order to identify statistical outliers, we applied the Nalimov outlier test. Statistical analyses were performed using OriginPro 8.6 (OriginLab Corp., Northampton, MA, USA). Statistical significance of a difference between two groups was tested using Student’s *t*-test. For multiple comparisons, one-way ANOVA and Fisher post hoc test were applied. A *P* < 0.05 was considered significant (*) and *P* < 0.001 was considered highly significant (***).

## Results

### Generation of macrophages during ARA affects vascular sprouting

**Fig. 1:**
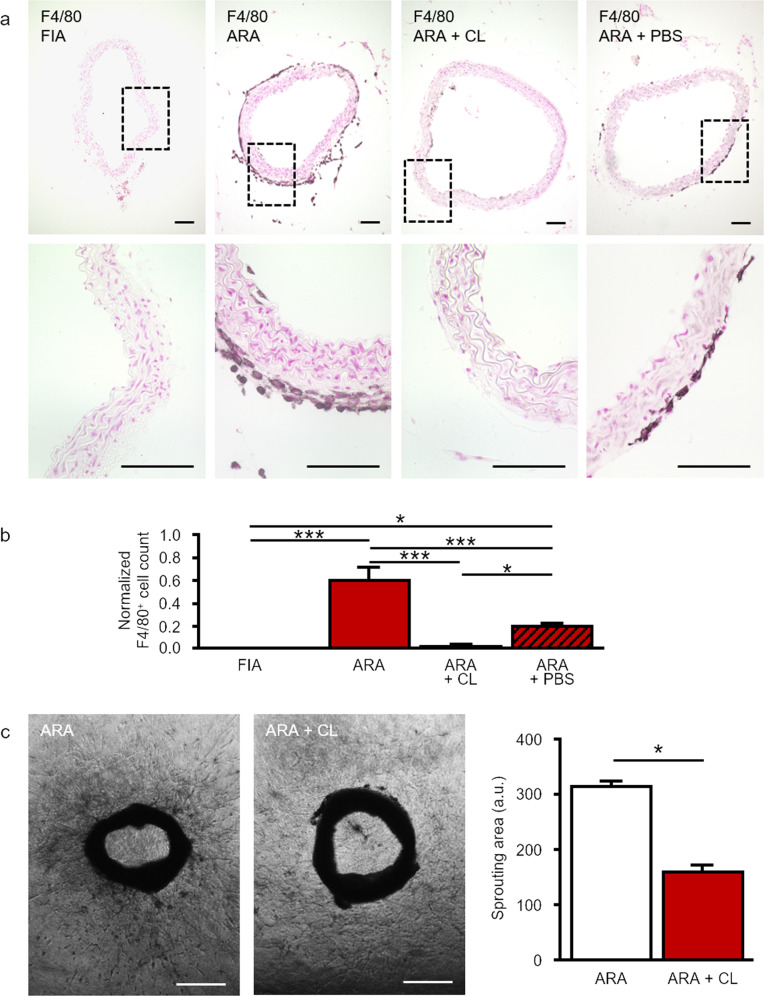
Generation of F4/80^+^ macrophages in ARA. **a** Cross-sections of murine aortae were immunostained for the marker of mature macrophages F4/80. In FIA, hardly any F4/80^+^ macrophage could be found. After ARA a few F4/80^+^ macrophages appeared within the intima. However, within the adventitia, the number of F4/80^+^ macrophages increased substantially. Compared to control, cultured aortic rings treated with clodronate-containing liposomes displayed almost no F4/80 immunostaining due to efficient depletion of F4/80^+^ macrophages within the adventitia. **b** Statistical analysis of the number of adventitial F4/80^+^ macrophages with and without liposome treatment. Application of PBS-containing liposomes had a less pronounced effect compared to clodronate-containing liposomes. Cell counts were normalized to total media area (*n* = 7–10). **c** Phase-contrast images and quantification of sprouting area of cultured aortic rings with or without clodronate treatment. Depletion of macrophages by clodronate-containing liposomes reduced total cellular sprouting as well as capillary-like tube formation (*n* = 23–31). *P < 0.05; ***P < 0.001. Scale bars: in **a** 100 µm, in **c** 500 µm. ARA aortic ring assay, ARA + CL ARA with clodronate-containing liposomes, ARA + PBS ARA with PBS-containing liposomes.

The presence of mature macrophages in cross-sections of FIA and after 11 days of ARA was analyzed by immunostaining for F4/80 [50, 51]. No macrophages could be detected within the different layers of FIA (Fig. 1a, b). However, after ARA F4/80^+^ cells were abundant especially within the aortic adventitia and occasionally also within the sub-endothelial space (Fig. 1a, b).

In order to assess the contribution of these locally generated macrophages to angiogenic sprouting, we depleted macrophages from aortic rings ARA by application of clodronate-containing liposomes as previously reported [45, 46]. Treatment of aortic rings with clodronate-containing liposomes efficiently depleted F4/80^+^ cells from the adventitia (Fig. 1a, b). This macrophage depletion in aortic rings reduced total cellular sprouting into the surrounding collagen gel during ARA by ~50% (Fig. 1c).

### Bone marrow-independent vascular progenitor cells contribute to macrophage generation in ARA

**Fig. 2:**
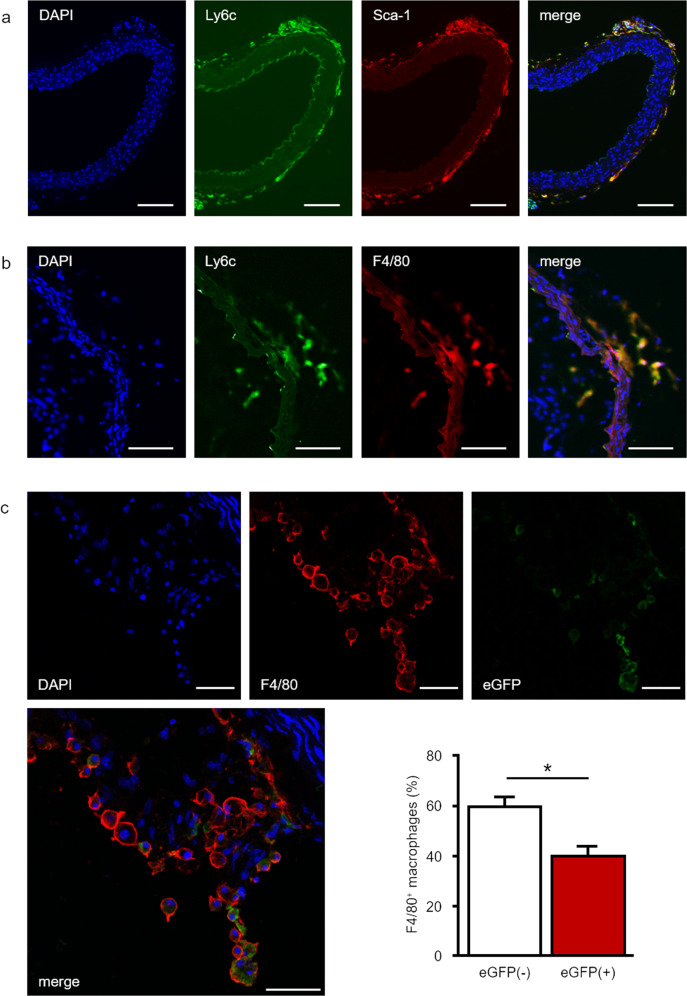
The majority of macrophages in ARA originates from bone marrow-independent progenitors. **a** Cross-sections of freshly isolated murine aortae were analyzed by immunofluorescence for the presence of Ly6c^+^ and Sca-1^+^ progenitor cells. Within the adventitia, a substantial part of Ly6c^+^ cells co-expressed Sca-1 distinguishing them from Ly6c^+^ monocytes that derived from the bone marrow and circulate in peripheral blood. **b** After ARA, cross-sections of murine aortae were analyzed by immunofluorescence for the presence of Ly6c^+^/F4/80^+^ cells. The majority of F4/80^+^ macrophages also expressed Ly6c suggesting their derivation from Ly6c^+^ progenitor cells. **c** Confocal image of a cross-section of a cultured aortic ring from the FlkSwitch mouse. These sections were screened for F4/80^+^ cells (red immunofluorescence) and eGFP+ cells (bone marrow-derived). Quantitative analysis of these sections (*n* = 6) confirmed that 40% of F4/80 macrophages originate from bone marrow-derived cells (F4/80^+^/eGFP^+^) whereas 60% are generated from bone marrow-independent progenitors (F4/80^+^/GFP^-^). Scale bars: in **a**, **b** 100 µm, in **c** 50 µm.

On cross-sections of FIA, we performed double immunofluorescence experiments in order to detect Ly6c^+^ and Sca-1^+^ cells. Within the sub-endothelial space, we identified Ly6c^+^ cells. However, these cells were negative for Sca-1 (Fig. 2a). In contrast, within the adventitia the majority of Ly6c^+^ cells co-expressed Sca-1 (Fig. 2a). Interestingly, we found that almost all F4/80^+^ adventitial macrophages generated during ARA also displayed immunoreactivity for Ly6c (Fig. 2b).

Next, aortic Ly6c^+^ cells were analyzed for their potential to differentiate into mature F4/80^+^ macrophages. To this end, Ly6c^+^ cells were isolated from FIA after tissue dispersion by means of Magnetic Activated Cell Sorting (MACS). Differentiation of the aortic Ly6c^+^ cell fraction was monitored by immunofluorescence staining for F4/80. At day 1 post sorting ~40% of cultured cells expressed F4/80. This fraction still increased until day 2 (Suppl. Fig. 1).

Bone marrow-derived cells, i.e., monocytes within the murine aortic wall, potentially give rise to macrophages generated in ARA. In order to test the contribution of these cells on macrophage generation during ARA, we used the transgenic FlkSwitch mouse model. In this model, all cells that differentiate from bone marrow-derived monocytes are labeled by eGFP expression. Analysis of cultured FlkSwitch aortic rings revealed that only 40% of F4/80^+^ macrophages expressed eGFP and therefore derived from bone marrow-dependent monocytes (Fig. 2c).

**Fig. 3:**
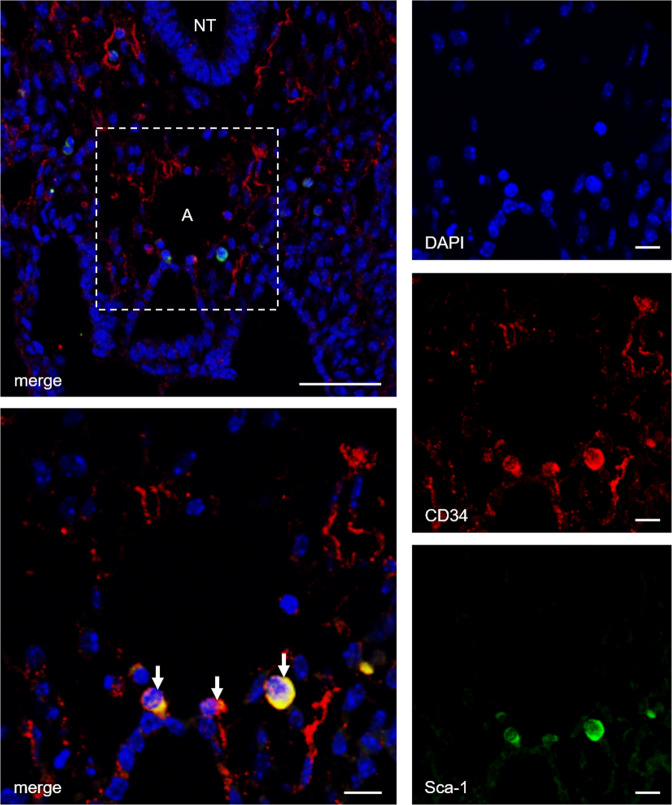
Aorta-associated progenitor cells may partially be yolk sac-independent. Confocal image of a transversal section from an *NCX1*^*−/−*^ mouse embryo (E9.5) immunostained for CD34 and Sca-1. The presence of CD34^+^/Sca-1^+^ cells surrounding the aortic region of these embryos that lack a functional vascular network to the yolk sac indicates a yolk sac-independent origin of these progenitor cells. Scale bars: lower magnification 50 µm, higher magnification 10 µm; NT neural tube, A aorta.

During embryonic development, the yolk sac represents a source for bone marrow-independent progenitor cells that can be found in different adult tissues. Therefore, we tested whether this also applies to adventitial Ly6c^+^/Sca-1^+^ progenitor cells. To this end, we stained embryonic tissue from *NCX1*^−*/*−^ mice that lack yolk sac perfusion for Ly6c^+^ and Sca-1^+^. Confocal microscopy revealed co-expression of Ly6c^+^/Sca-1^+^ in cells surrounding the embryonic aortic wall (Fig. 3).

### Locally generated macrophages promote activation and proliferation of VW-SCs

Next, we investigated the contribution of locally generated macrophages on the mitotic activity of vascular cells during ARA. To address this issue, we analyzed cross-sections of FIA and cultured aortic rings for the proliferation marker Ki67 [52, 53]. Compared to FIA, the number of proliferating cells substantially increased during ARA especially within the adventitia (Suppl. Fig. 2). Clodronate-mediated macrophage depletion completely prevented the increase in Ki67^+^ cells during ARA (Suppl. Fig. 2).

**Fig. 4:**
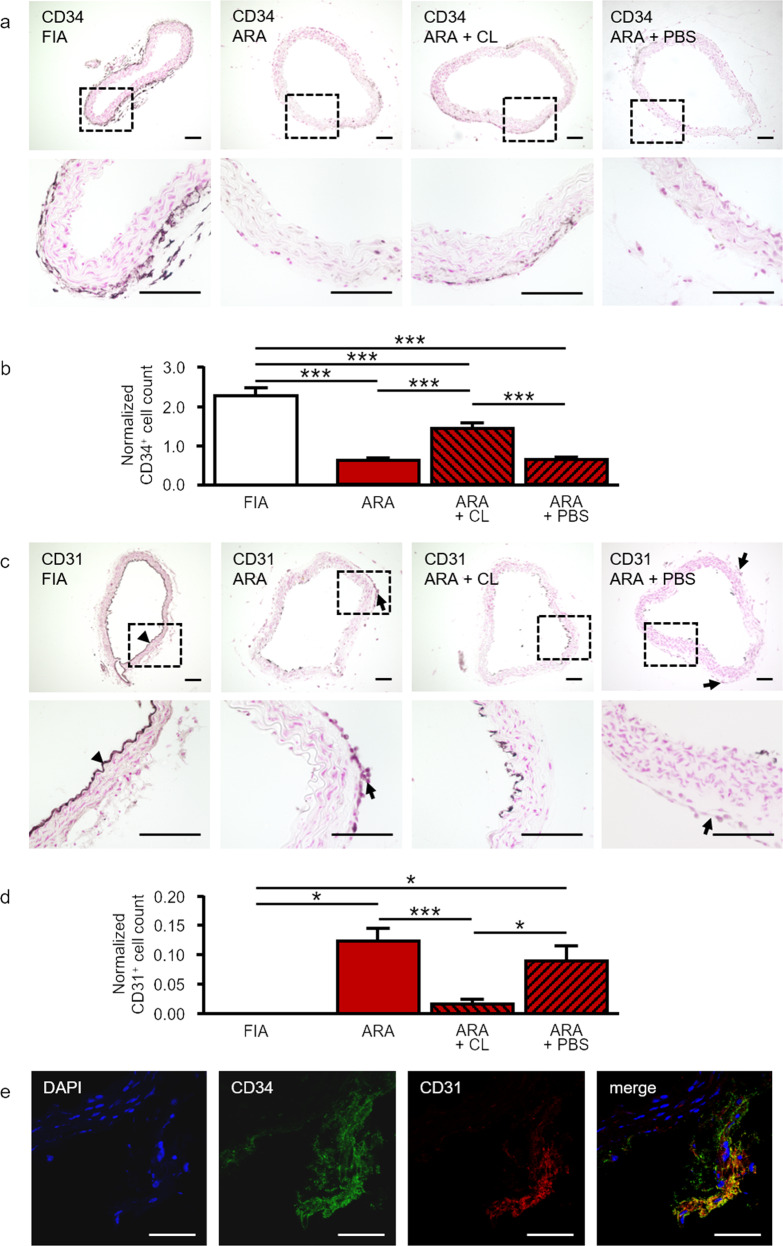
Macrophage depletion prevents activation of the vasculogenic zone and generation of CD31^+^ endothelial cells in ARA. **a** Cross-sections of murine aortae were immunostained for the progenitor cell marker CD34. In FIA, a large number of CD34^+^ progenitor cells was present within the vasculogenic zone of the adventitia. By performing ARA, these cells were activated within their adventitial stem cell niche and differentiated thereby losing their CD34 immunoreactivity. Depletion of macrophages preserved the number of CD34^+^ cells within the adventitia during ARA. **b** Statistical analysis of the number of adventitial CD34^+^ cells with and without liposome treatment. Cell counts were normalized to the total media area (*n* = 6–12). **c** Cross-sections of murine aortae were immunostained for the endothelial cell marker CD31. In FIA, CD31^+^ cells were present within the intima as expected (arrowhead), but were almost completely absent from the adventitia. After ARA, a substantial number of CD31^+^ cells appeared within the adventitia (arrows). Their generation was largely prevented by the depletion of macrophages. **d** Statistical analysis of the number of adventitial CD31^+^ cells with and without liposome treatment. Cell counts were normalized to total media area (*n* = 3–10). **e** Cross-section of a cultured aortic ring at day 3 immunostained for CD34 and CD31. CD34^+^/CD31^+^ cells represent a transitional stage in the differentiation of CD34^+^ adventitial progenitors towards CD31^+^ endothelial cells. *P < 0.05; ***P < 0.001. Scale bars: in **a** and **c** 100 µm, in **e** 50 µm. FIA fresh isolated aorta, ARA aortic ring assay, ARA + CL ARA with clodronate-containing liposomes, ARA + PBS ARA with PBS-containing liposomes.

Furthermore, we sought to analyze the impact of locally generated macrophages on the adventitial stem cell niche, the vasculogenic zone. In FIA, this zone contains a high number of CD34^+^ progenitor cells within the entire circumference of the adventitial layer (Fig. 4a). Immunohistochemical analyses showed a distinct reduction of these CD34^+^ cells during ARA (Fig. 4a, b). This may indicate the initiation of a differentiation process that is accompanied by a down-regulation of CD34 expression in these cells. In contrast, macrophage depletion significantly preserved the number of CD34^+^ cells within the adventitial vasculogenic zone (Fig. 4a, b).

As expected, in FIA CD31^+^ endothelial cells were only present within the intima. In contrast to CD34, almost no CD31^+^ cells could be found within the adventitia of FIA (Fig. 4c, d). However, after ARA the number of CD31^+^ endothelial cells was increased considerably within the adventitia (Fig. 4c, d). Again, macrophage depletion by clodronate almost completely attenuated the generation of CD31^+^ cells from the adventitial progenitors (Fig. 4c, d). The impaired generation of CD31^+^ cells within the adventitia upon clodronate treatment was not attributable to a direct cytotoxic effect of clodronate on CD31^+^ cells, since the number of CD31^+^ cells within the endothelium was not changed upon this treatment (Suppl. Fig. 3a). Furthermore, direct incubation with clodronate-containing liposomes did not affect the cell viability of cultured EA.hy926 endothelial cells (Suppl. Fig. 3b).

Reciprocal alterations in the number of CD34^+^ and CD31^+^ cells during ARA may suggest the coupling of both phenomena. Therefore, we analyzed cross-sections of cultured aortic rings on day 3. Confocal immunofluorescence analyses demonstrated CD34^+^/CD31^+^ co-expressing cells representing a transitional stage in the differentiation process (Fig. 4e).

### Locally generated macrophages are the source and target of VEGF in ARA

**Fig. 5:**
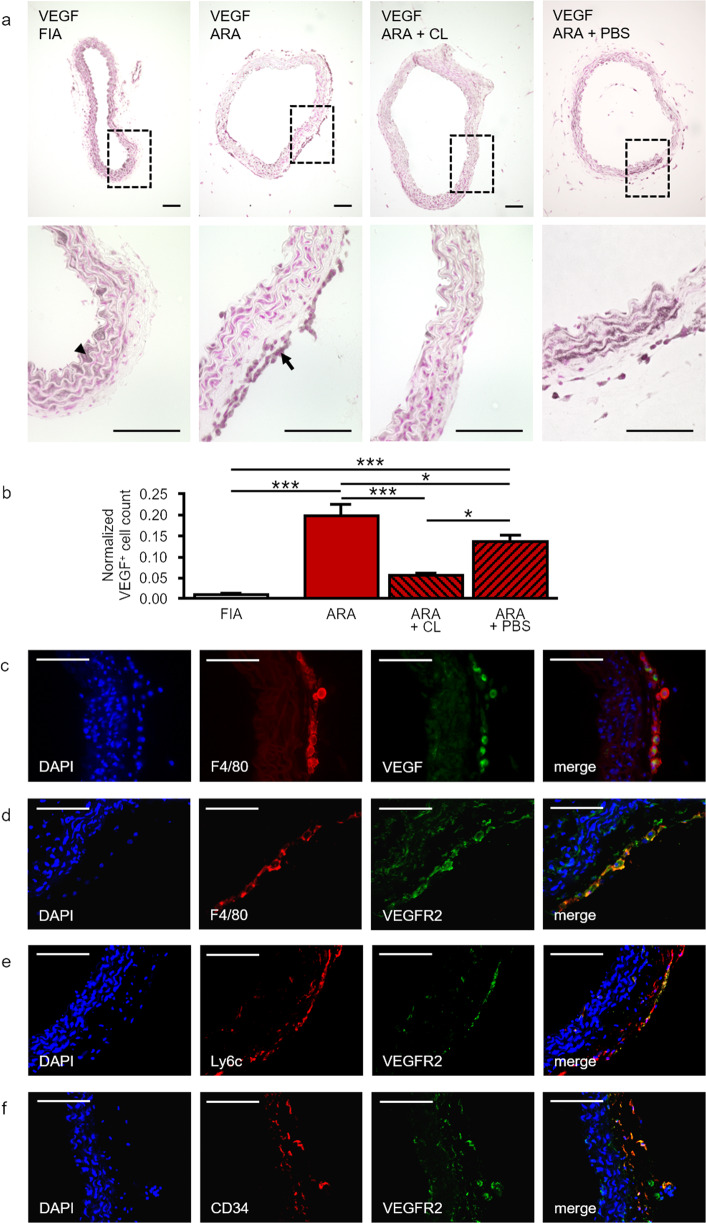
Expression of VEGF/ VEGFR2 in macrophages and CD34^+^ progenitor cells in ARA. **a** Cross-sections of murine aortae were immunostained for the pro-angiogenic factor VEGF. In FIA, VEGF is predominantly expressed by intimal cells (arrowhead) and cells within the media. In ARA the number of VEGF^+^ cells increased substantially especially within the adventitia (arrow). This increase was largely prevented by the depletion of macrophages. **b** Statistical analysis of the number of adventitial VEGF^+^ cells with and without liposome treatment. Cell counts were normalized to total media area (*n* = 6–8). **c** Cross-sections of cultured aortic rings were analyzed by confocal co-immunofluorescence for the presence of VEGF^+^ and F4/80^+^ cells, respectively. Almost all adventitial cells that were highly positive for VEGF co-express F4/80. **d** Additionally, F4/80^+^ cells in cross-sections of cultured aortic rings co-express VEGFR2. **e**, **f** Similarly, in cross-sections of FIA Ly6c^+^ and CD34^+^ progenitor cells show at least partially co-expression of VEGFR2 by confocal immunofluorescence microscopy. *P < 0.05; ***P < 0.001. Scale bars: 100 µm. FIA fresh isolated aorta, ARA aortic ring assay, ARA + CL ARA with clodronate-containing liposomes, ARA + PBS ARA with PBS-containing liposomes.

Macrophages were shown to secrete high levels of VEGF under pathological conditions, i.e., in atherosclerosis, under hypoxia, and in tumors [54, 55]. In FIA, immunostaining revealed low VEGF expression in a small fraction of sub-endothelial and adventitial cells (Fig. 5a, b). In contrast, after ARA augmented VEGF immunostaining was detected in adventitial cells (Fig. 5a, b). The distribution pattern of VEGF^+^ cells (Fig. 5a) resembled that of F4/80^+^ cells (Fig. 1a). Moreover, macrophage depletion almost abolished VEGF^+^ cells from cultured aortic rings (Fig. 5a, b). Therefore, we conducted co-immunofluorescence staining for F4/80 and VEGF. Confocal analyses demonstrated that the majority of adventitial VEGF^+^ cells also expressed the macrophage marker F4/80 (Fig. 5c) identifying adventitia-derived macrophages as the main source of pro-angiogenic VEGF in ARA. Furthermore, F4/80^+^ macrophages, as well as Ly6c^+^ and CD34^+^ progenitor cells are VEGF responsive, since they also express the VEGFR2 (Fig. 5d–f).

**Fig. 6:**
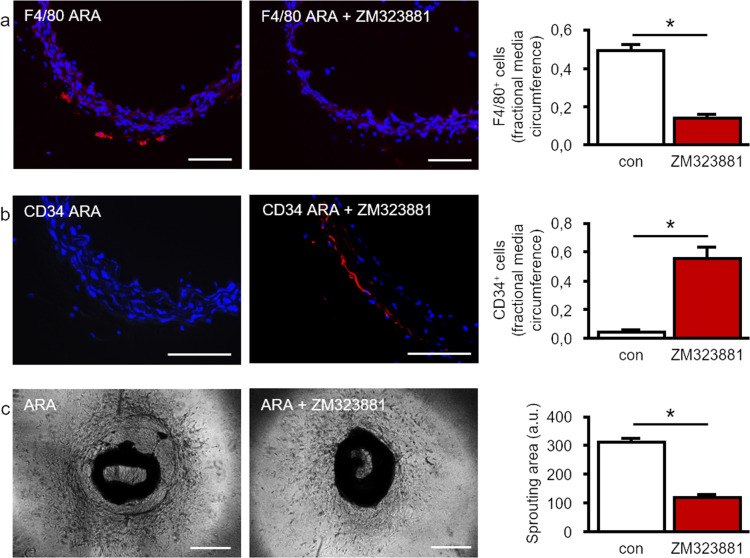
Macrophage-derived VEGF activates the vasculogenic zone and promotes macrophage generation. **a** After ARA, a substantial number of F4/80^+^ macrophages was generated whereas hardly any macrophage was detected after ARA in the presence of the VEGFR2 antagonist ZM323881 (1 µM). For quantification, the fraction of media circumference that is covered by F4/80^+^ cells was analyzed (*n* ≥ 5). **b** Furthermore, the application of ZM323881 (1 µM) resulted in the preservation of CD34^+^ cells within the adventitial vasculogenic zone. For quantification, the fraction of media circumference that is covered by CD34^+^ cells was analyzed (*n* ≥ 4). **c** Consequently, cellular sprouting, as well as capillary-like tube formation in ARA, were reduced in the presence of ZM323881 (1 µM; *n* = 31). *P < 0.05. Scale bars: 100 µm, phase contrast 500 µm. ARA aortic ring assay.

Finally, we tested whether VEGF produced by locally generated macrophages affects their own generation and contributes to the remodeling of the adventitial vasculogenic zone during ARA. To this end, we used the specific VEGFR2 antagonist ZM323881. As shown in Fig. 6a, pharmacological inhibition of VEGFR2 signaling distinctly impaired the generation of F4/80^+^ macrophages within the adventitia. Furthermore, ZM323881 preserved the presence of CD34^+^ cells within the vasculogenic zone of cultured aortic rings (Fig. 6b). This impaired activation of the vasculogenic zone upon VEGFR2 blockage consequently resulted in reduced capillary-like tube formation as well as total cellular sprouting (Fig. 6c).

## Discussion

For decades, macrophages were believed to originate exclusively from bone marrow-derived monocytes that had infiltrated the vessel wall from the circulation [56]. In this study, we aimed to prove the role of bone marrow-independent VW-SC-derived macrophages in new vessel formation by angiogenesis and/or postnatal vasculogenesis. For this purpose, we made use of the murine ARA. In contrast to cell culture experiments, this ex vivo assay enables in situ investigation of the complex interplay of different vascular wall cell types in angiogenesis by the cultivation of the entire vessel wall [42–44].

Our results demonstrate that: (I) F4/80^+^ macrophages generated during ARA predominantly originate from bone marrow-independent and at least partially yolk sac-independent VW-SCs residing within the aortic adventitia; (II) these macrophages are depleted by clodronate treatment indicating a phagocytic capacity similar to bone marrow-derived macrophages; (III) macrophages represent the main source of VEGF within the angiogenically activated vessel wall; (IV) VEGF stimulates further macrophage generation in an auto- and paracrine manner leading to a pro-angiogenetic feedforward loop; (V) macrophage depletion as well as blockage of VEGF/VEGFR2 signaling resulted in the preservation of the stem cell niche within the adventitial vasculogenic zone and impaired sprouting activity. These data show that bone marrow-independent macrophages generated from adventitial VW-SCs substantially contribute to the control of the adventitial stem cells niche and new vessel formation via VEGF/VEGFR2 signaling.

Generation of macrophages from cells within the aorta was previously shown using mouse and rat aorta as well as human internal thoracic artery [34, 44, 39]. These data were later confirmed by Psaltis et al. who showed that Sca-1^+^/CD45^+^ progenitor cells within the adventitia are capable to differentiate into macrophages in vitro [29]. However, the definite origin of these progenitor cells, bone marrow-derived or adventitia-resident, remained uncertain. Our study substantiates the concept of bone marrow-independent generation of macrophages from local resident progenitor cells and underscores their importance to angiogenetic processes.

Similar to Psaltis et al. [29], we demonstrate here that in cultured aortic rings adventitia-resident Ly6c^+^ cells give rise to mature F4/80^+^ macrophages in situ. Since circulating and tissue-infiltrated monocytes also express Ly6c [57], this could not rule out that all detected macrophages in cultured aortic rings originate from previously infiltrated bone marrow-derived monocytes. However, it was shown that bone marrow-derived monocytes lose their Sca-1 expression before entering the circulation [58]. Because we found that the vast majority of adventitial Ly6c^+^ cells co-express Sca-1 it is highly unlikely that the majority of adventitial F4/80^+^ macrophages in cultured aortic rings originate from bone marrow-derived monocytes. This conclusion is further supported by ARA experiments using the FlkSwitch reporter mouse [59, 60]. This genetic mouse model enables to identify cells that originate from bone marrow. Only cells derived from bone marrow, e.g., monocytes, are permanently labeled by switching expression from red-fluorescent tdTomato protein to green-fluorescent eGFP. Performing ARA with aortic rings from FlkSwitch mice, we found that ~60% of macrophages generated in cultured aortic rings do not originate from bone marrow-derived cells. These data suggest that the contribution of bone marrow-independent local progenitors to the generation of vascular wall macrophages during angiogenic activation is much more important than previously assumed.

These bone marrow-independent macrophage progenitors within the vascular wall might originate from the yolk sac during embryonic development as shown for definitive macrophages in different organs [61, 62]. We hypothesized that a progenitor cell population surrounding the embryonic aorta persists until the adult vascular adventitia. To test this hypothesis, we analyzed transversal sections of embryos at E9.5 of the *NCX1*^*−/−*^ mouse model. At that early stage of embryonic development, progenitor cells cannot originate from bone marrow. Moreover, these *NCX1*^*−/*−^ mice show impaired embryonic circulation due to the absence of cardiac beating leading to lethality in utero [41]. Additionally, although yolk sac-dependent hematopoiesis appears to be regular in these mice, they do not develop a perfusable vascular system within the yolk sac [40]. Since yolk sac-derived cells are known to migrate into the embryo via the vascular system [62], it is highly unlikely that the periaortic Ly6c^+^/Sca-1^+^ progenitor cells we observed in *NCX1*^*−/*−^ mouse embryos originate from the yolk sac. This suggests that at least a part of the Ly6c^+^/Sca-1^+^ macrophage progenitor cells within the vascular adventitia neither derives from bone marrow nor from the yolk sac but from periaortic stem cells during embryonic development.

Angiogenic activation in ARA is characterized by distinct alterations, i.e., the proliferation of cells greatly contributes to vascular sprouting and new vessel formation. Accordingly, we found an increased number of proliferating Ki67^+^ cells in cultured aortic rings. Furthermore, angiogenesis depends on differentiation processes. Previously, our group demonstrated the presence of an adventitial vasculogenic zone that contains stem and progenitor cells capable to differentiate into vascular and non-vascular cell types [34, 63]. The majority of these VW-SCs are CD34^+^ [34, 63]. Therefore, the number of CD34^+^ cells during ARA is an indicator of activation of this vasculogenic zone. We found that during ARA the number of these CD34^+^ cells drastically declined. Conversely, CD31^+^ cells representing endothelial cells or their progenitors [64] could not be detected within the adventitia of FIA but appeared during ARA. The coincidence suggested that CD31^+^ cells might differentiate from CD34^+^. This assumption was verified by the presence of CD34^+^/CD31^+^ cells at day 3 of ARA representing a transitional stage during differentiation.

Next, we aimed to analyze the contribution of locally generated macrophages to the proliferation and differentiation of cells during ARA. To this end, we depleted macrophages from ARA by application of clodronate-containing liposomes [45, 46]. Interestingly, the increase in Ki67^+^ cells, the decline in CD34^+^ cells, and the appearance of CD31^+^ cells during ARA were all significantly reduced by macrophage depletion. Accordingly, capillary-like tube formation, as well as total cellular sprouting were substantially reduced. This clearly indicates a crucial role of these locally generated macrophages in angiogenesis. Similar to previously published data [65], clodronate-free liposomes also affected the number of macrophages and sprouting cells. However, this effect was significantly less pronounced compared to clodronate-containing liposomes.

**Fig. 7:**
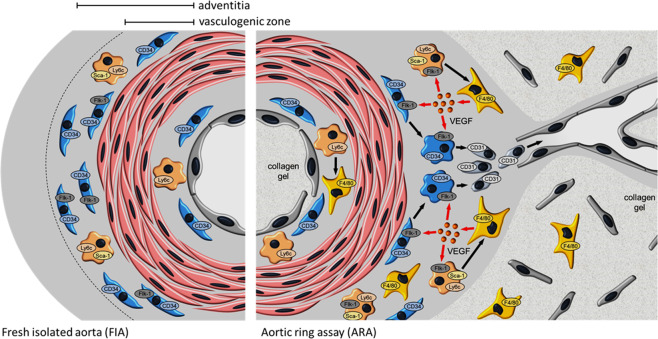
Interplay of adventitial cells in angiogenic activation. In ARA the majority of macrophages arise from resident bone marrow-independent and at least partially yolk sac-independent Ly6c^+^/Sca-1^+^ adventitial progenitor cells. VEGF released by macrophages on the one hand induces angiogenetic sprouting by stimulating the differentiation of CD34^+^ progenitor cells within the adventitial vasculogenic zone into CD31^+^ endothelial cells. On the other hand, macrophage-derived VEGF potentiates the generation of macrophages via VEGF/VEGFR2 signaling in terms of a feedforward loop thereby maintaining the angiogenic process.

Since depletion of macrophages almost completely attenuated the proliferation and differentiation activity within the adventitia, we hypothesized that a soluble factor secreted by macrophages may be involved. We found that F4/80^+^ macrophages generated during ARA highly express VEGF. They become the main source of this potent pro-angiogenic factor. This is in accordance with previous reports on VEGF secretion by macrophages [54, 55]. In order to prove the functional relevance of macrophage-derived VEGF in ARA, we pharmacologically blocked VEGF/VEGFR2 signaling using the specific VEGFR2 antagonist ZM323881. Similar to macrophage depletion, this intervention interfered with the activation of the vasculogenic zone as indicated by preserved numbers of CD34^+^ cells, and hence reduced vascular sprouting activity. Moreover, VEGFR2 antagonism diminished the appearance of F4/80^+^ cells during ARA suggesting a vicious cycle of macrophage generation. This view is further supported by the expression of VEGFR2 on F4/80^+^ macrophages as well as on Ly6c^+^ progenitor cells. This feedforward loop driven by the interrelation between macrophages and VEGF maintains a pro-angiogenic microenvironment that promotes proliferation and differentiation of VW-SCs into vascular cells necessary for capillary sprouting (Fig. 7).

Our data on the origin and role of macrophages in angiogenesis may be of great clinical relevance. In cancer, TAMs mediate tumor growth, angiogenesis, and invasion in a VEGF-dependent manner. It is not known whether TAMs differentiate from monocytes recruited from the circulation or from resident progenitor cells [14]. Our findings argue in favor of a critical contribution of bone marrow-independent macrophages and may explain the low efficacy of monocyte recruitment inhibition in clinical studies on cancer therapy [22–28].

Furthermore, our results may also be relevant for chronic diseases with long-lasting pro-inflammatory activation, i.e., atherosclerosis. Interestingly, Robbins et al. showed that macrophage accumulation in the atherosclerotic plaque is rather due to the proliferation of local macrophages than caused by infiltrated monocyte [66] and VEGF was shown to play a crucial role in atherosclerosis, too [67, 68]. According to our results, VEGF-driven differentiation of VW-SCs into macrophages could represent the starting point for the subsequent proliferation of macrophages. Further studies are needed to prove the role of VW-SC-derived macrophages in such diseases and to identify potential targets for new therapeutic strategies.

## Supplementary information

Supplemental Figure 1

Supplemental Figure 2

Supplemental Figure 3

Supplemental Figure Legends

aj-checklist

## Data Availability

Data were available upon request from the authors.
