# Supplementary figures and images for: Bone marrow-independent adventitial macrophage progenitor cells contribute to angiogenesis
Source: Cell Death Dis. 2022 Mar 9;13(3):220. doi: 10.1038/s41419-022-04605-2 (PMC8907187; doi:10.1038/s41419-022-04605-2)

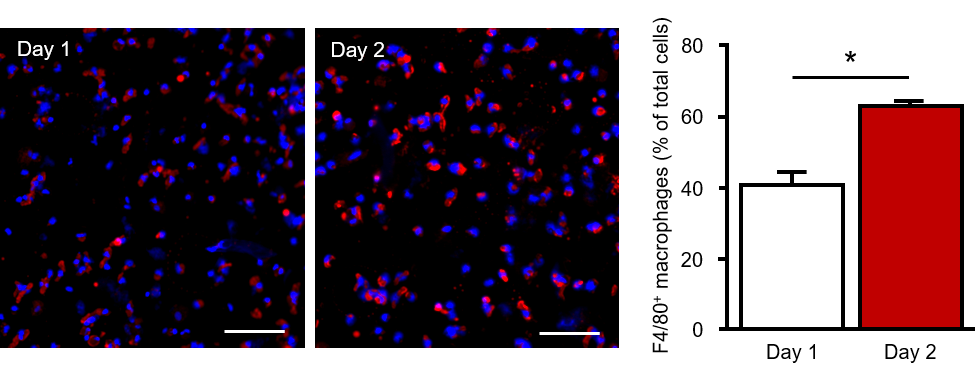

Supplement: Supplementary file 1 — Supplemental Figure 1 [file 41419_2022_4605_MOESM1_ESM.tif]

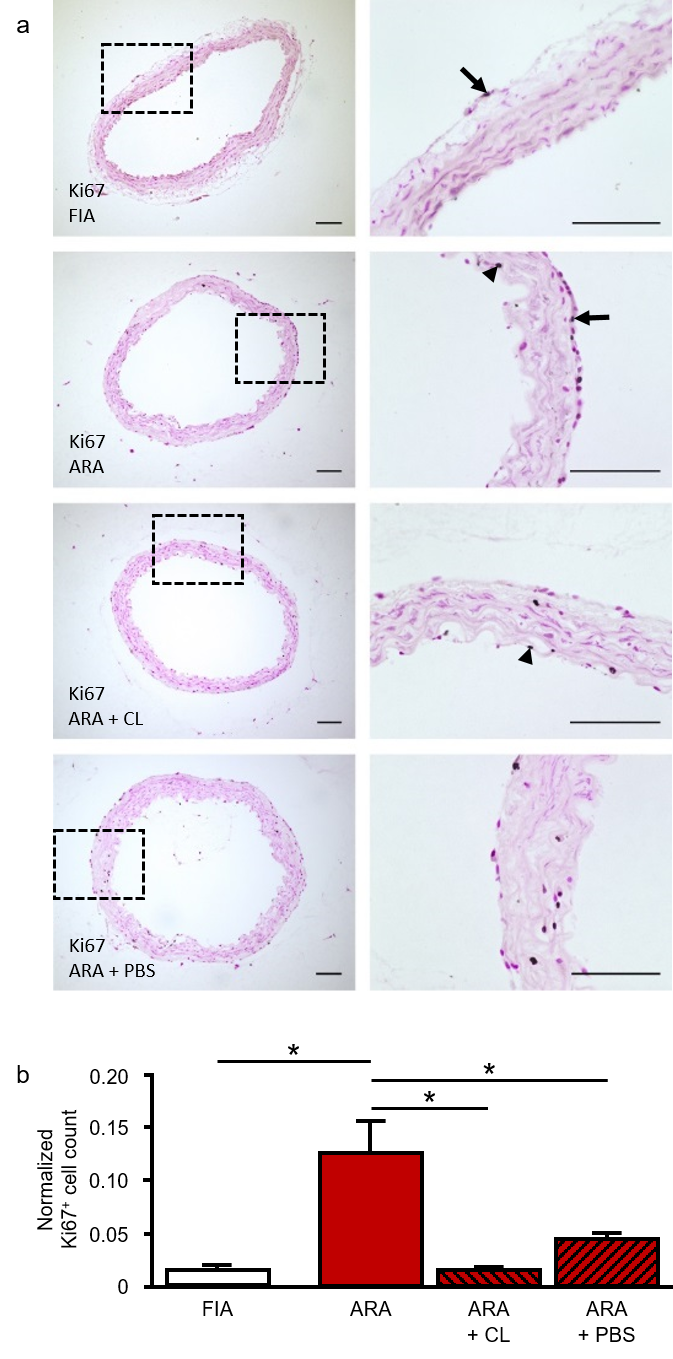

Supplement: Supplementary file 2 — Supplemental Figure 2 [file 41419_2022_4605_MOESM2_ESM.tif]

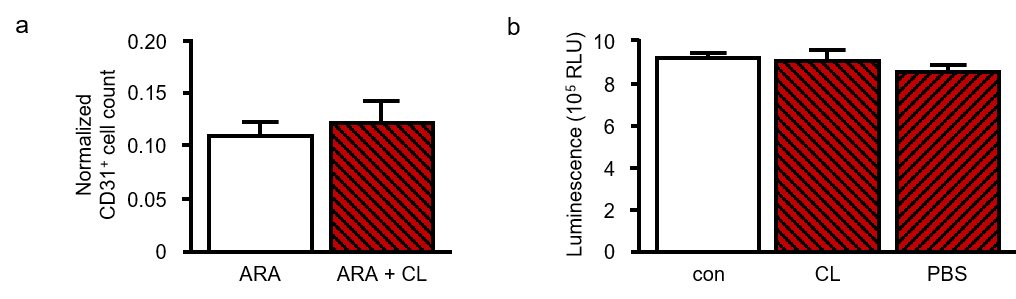

Supplement: Supplementary file 3 — Supplemental Figure 3 [file 41419_2022_4605_MOESM3_ESM.tif]
