# Supplementary material for: Bone marrow-independent adventitial macrophage progenitor cells contribute to angiogenesis
Source: Cell Death Dis. 2022 Mar 9;13(3):220. doi: 10.1038/s41419-022-04605-2 (PMC8907187; doi:10.1038/s41419-022-04605-2)
Supplement: Supplementary file 4 — Supplemental Figure Legends [file 41419_2022_4605_MOESM4_ESM.docx]

**Supplement Figure S1. Ly6c^+^ cells differentiate into F4/80^+^ macrophages.**

Ly6c^+^ cells were isolated from murine aortae, sorted by MACS and subsequently cultured for three days. Immunofluorescence analysis showed an increasing fraction of F4/80^+^ cells (red fluorescence). For quantification, F4/80^+^ cells are given as the percentage of total cell count (n=3). Scale bars: 100 µm.

**Supplement Figure S2. Macrophages promote proliferation in ARA.**

(a) Cross-sections of murine aortae were immunostained for the proliferation marker Ki67. Compared to FIA, the number of Ki67^+^ cells increased after ARA. Depletion of macrophages by application of clodronate resulted in an almost complete disappearance of Ki67^+^ cells in cultured aortic rings. (b) Statistical analysis of the number of adventitial Ki67^+^ cells with and without liposome treatment. Cell counts were normalized to total media area (n=3-5). *P<0.05. Scale bars: 100 µm. FIA: fresh isolated aorta; ARA: aortic ring assay; ARA+CL: ARA with clodronate-containing liposomes; ARA+PBS: ARA with PBS-containing liposomes.

**Supplement Figure S3. Clodronate is not toxic to endothelial cells.**

(a) In ARA, the number of CD31^+^ cells within the endothelium was not altered by clodronate treatment excluding a direct cytotoxic effect on CD31^+^ endothelial cells. Cell counts were normalized to total media area (n=10). (b) Cell viability of EA.hy926 endothelial cells was determined using a luminescent ATP detection assay. Cells were left untreated or were incubated with clodronate-containing liposomes (CL) or PBS-containing liposomes (PBS) for 48h. These incubations had no toxic effect on endothelial cells (n=7).
